# The Impact of Ageing on the CNS Immune Response in Alzheimer’s Disease

**DOI:** 10.3389/fimmu.2021.738511

**Published:** 2021-09-17

**Authors:** Stephan En Jie Chee, Egle Solito

**Affiliations:** ^1^Barts and The London School of Medicine and Dentistry, Queen Mary University of London, London, United Kingdom; ^2^William Harvey Research Institute, Barts and The London School of Medicine and Dentistry, Queen Mary University of London, London, United Kingdom; ^3^Dipartimento di Medicina Molecolare e Biotecnologie Mediche, Federico II University, Naples, Italy

**Keywords:** immunosenescence, Alzheimer, neuroinflammation, biomarker, screening, aging

## Abstract

Alzheimer’s Disease (AD) is a progressive neurodegenerative disease strongly associated with increasing age. Neuroinflammation and the accumulation of amyloid protein are amongst the hallmarks of this disease and most translational research to date has focused on targeting these two processes. However, the exact etiology of AD remains to be fully elucidated. When compared alongside, the immune response in AD closely resembles the central nervous system (CNS) immune changes seen in elderly individuals. It is possible that AD is a pathological consequence of an aged immune system secondary to chronic stimulation by a previous or ongoing insult. Pathological changes like amyloid accumulation and neuronal cell death may reflect this process of immunosenescence as the CNS immune system fails to maintain homeostasis in the CNS. It is likely that future treatments designed to modulate the aged immune system may prove beneficial in altering the disease course. The development of new tests for appropriate biomarkers would also be essential in screening for patients most likely to benefit from such treatments.

## Introduction

### Epidemiology

Alzheimer’s disease (AD) is a progressive neurodegenerative disease characterized clinically by memory and cognitive impairments initially, and further neurocognitive deterioration with time (1). It is the most common cause of dementia, estimated to account for 60-70% of cases worldwide (2), and is strongly associated with increasing age, with about 10% of people aged 65 years and over and 32% of those over 85 years of age in the US having AD (2). Most cases are late onset AD (LOAD) and albeit being associated with various risk factors, most prominently age, family history in a first degree relative and specific genotypes, they are considered sporadic and idiopathic (1). Other types of AD include familial AD, early onset AD (EAOD) and AD associated with Down’s syndrome (DS), although these types are rare and constitute only a small proportion of cases (1).

### Pathological Features of AD and the Aging CNS

Some of the classical hallmarks of AD pathology include amyloid β (Aβ) accumulation and neurofibrillary pathology. Aβ monomers are derived from the cleavage of amyloid precursor protein (APP), a membrane protein concentrated in neurons in the brain and whose function remains to be elucidated (3, 4). These monomers then aggregate to form Aβ oligomers which in turn form insoluble amyloid plaques extracellularly which are neurotoxic (5). It is worth noting that whilst much attention has traditionally been given to amyloid plaques, Aβ oligomers are now considered to be the most neurotoxic form (6).

Another hallmark of AD is the neurofibrillary pathology due to abnormal aggregation of tau protein. These proteins are normally modified physiologically to stabilize microtubules, coordinate axonal transport and maintain DNA integrity (5). As such, the pathological modification of tau in AD can lead to impaired neuronal health and loss (5).

Nine hallmarks of aging that characterizes the aging process have been described by Lopez-Otin et al. (7). Of particular note is the loss of proteostasis, whereby the various mechanisms that normally ensures the stabilization of correctly folded proteins and the degradation and removal of misfolded proteins are altered (7). For example, age-related LOAD has been demonstrated to have higher than normal expression of the chaperone heat-shock protein (HSP) 27 in degenerating areas of AD brains, and that this is thought to be a compensatory response to a proteotoxic CNS environment (8–11). Another important hallmark of note is altered intercellular communication, which includes changes in immunosurveillance and an inflammatory state resulting in “inflammaging” (7). For example, inflammaging can arise from various causes such as the accumulation of proinflammatory tissue damage and an increasingly dysfunctional immune system for maintaining homeostasis, resulting in the upregulation of pro-inflammatory pathways such as the NLRP3 inflammasome (12). As Aβ accumulation and neuroinflammation are amongst the key pathological features of AD, such age-related hallmarks suggest that AD pathogenesis might reflect a pathological consequence of the normal aging process.

## CNS Immune Response in AD

### Microglia

One immune cell type within the CNS that has been the subject of much research into neurodegenerative disease is the microglia. These are tissue-resident macrophages that are epigenetically and functionally different to peripheral monocytes and macrophages, and play an essential role in maintaining neuronal health and homeostasis. In the resting state, microglia extend various processes that allow it to monitor the neuronal environment, and alter their morphology and gene expression in response to different stimuli (13). Such stimuli include damage-associated molecular patterns (DAMPs) and pathogen-associated molecular patterns (PAMPs), ATP released by neuronal damage, cell debris and abnormal proteins such as Aβ amyloid (5, 13). The mechanisms employed by activated microglia to these stimuli are diverse, ranging from phagocytosis to the release of numerous cytokines and chemokines (14, 15). Given that microglia constitute part of the CNS immune system, AD pathogenesis has been suggested and traditionally believed to be due to inappropriate neuroinflammation. For example, microglia are commonly observed aggregating around Aβ plaques in response to chemotaxis from Aβ deposits (16). In addition, these microglia express increased levels of reactive oxygen species (ROS) production that inherently damage surrounding neurons. These activated microglia also express upregulated major histocompatibility complex (MHC) class II levels and increased secretion of pro-inflammatory cytokines interleukin (IL)-1β, IL-6, tumor necrosis factor (TNF) and IL-8 (17, 18). Such findings support the theory that microglia may have a pro-inflammatory role in the perpetuation of AD.

However, as mentioned previously, microglia also play an important role in maintaining homeostasis in the neuronal environment and it has increasingly been suggested that AD pathogenesis occurs secondary to microglial dysfunction. For example, microglial dystrophy is a morphologically distinct phenotype to that of activated and resting microglia and includes features such as cytoplasmic fragmentation (19). This is characteristic of microglia which are abundant in areas of the AD brain showing high levels of neurofibrillary pathology (19). It is also worth noting that different microglial phenotypes exist in AD which are associated with and reflect an accelerated aging process and direct phagocytosis of Aβ plaques (20). This has led to the hypothesis that the AD microenvironment accelerates the transcriptional trajectory of aging microglia, but which can be altered upon phagocytosis of synaptic components around Aβ plaques – a process mediated by and reinforced by the *Hif1a* regulon (20).

In addition, the presence of these dystrophic microglia has been observed to precede that of the neuritic plaques (19). One manner which microglia may contribute to these plaques is through the inflammasome-mediated recruitment of the adaptor protein apoptosis-associated speck-like protein containing a CARD (ASC) (21). For example, ASC was found to promote Aβ aggregation in AD patients and mice models, and to promote Aβ deposition in mice models of AD (21).

Brain regions showing elevated levels of dysmorphic microglia have also been observed to have increased cellular debris accompanied by an absence of brain macrophages, suggesting that microglial dystrophy might indicate an impairment of microglial phagocytic function and possibly microglial loss (22). It has also been shown that microglia play an essential role in synaptic pruning through various mechanisms like modifying the perisynaptic environment, remodeling and phagocytosing dendritic spines and axon terminals, and coordinates closely with astrocytes in synaptic pruning (23, 24). As such, microglial phagocytic dysfunction might also adversely affect synaptic pruning. It is also well-documented that neuronal synapse loss occurring early in AD is due to dysfunctional complement-mediated pruning of synapses by microglia and that this is strongly correlated with cognitive decline (25, 26).

Aβ deposits have also been observed to accumulate in the absence of microglia in transgenic mice, suggesting the role of microglia in removing Aβ deposits (27). As such, it has been postulated that microglial neurodegeneration results in the disturbed neuronal environment of tau and Aβ accumulation characteristic of AD pathophysiology (28).

### Astrocytes

Another CNS immune cell type that has been implicated in AD is the astrocyte. Similar to microglia, astrocytes normally serve to maintain homeostasis within the neuronal environment. Some of the functions include maintaining the integrity of neuronal synapses *via* neurotrophic and metabolic support, maintaining the blood-brain barrier (BBB) with endothelial cells as part of the neurovascular unit (NVU), regulating cerebral blood flow, and removing neurotoxic waste products such as tau and amyloid *via* the glymphatic system (5, 18). Astrocytes are also attracted to and migrate towards Aβ deposits with evidence that there are attempts by these cells to remove them (5). The findings that Aβ has been found in astrocytes around Aβ deposits and that lower Aβ levels are associated with increased local astrocyte population suggest an active role played by astrocytes in the removal and degradation of Aβ deposits (29, 30). They have also been reported to adopt a pro-inflammatory phenotype in response to Aβ deposits, characterized by the release of various pro-inflammatory cytokines similar to those produced by activated microglia, a loss of their homeostatic function and an ability to induce apoptosis in neurons and oligodendrocytes (18, 31). As such, similarly to microglia, either an inappropriate neuroinflammatory response by astrocytes or astrocytic dysfunction may contribute to the pathogenesis of AD. It is also worth noting that astrocytes that accumulate Aβ can lyse to produce Aβ plaques, likely potentiating the pathological course of AD (32). It is also worth noting that IL-3 produced by astrocytes has recently been implicated as potentially having protective benefits in AD, with increased Aβ burden and worsening of memory impairment being recorded in knockout mice models of AD (33).

The role of astrocytes in maintaining the BBB and the glymphatic system has in recent years been particularly explored as a key contributor to AD pathogenesis. One of the features of AD is an impairment of the BBB, which is associated with reduced cerebral blood flow. This cerebrovascular dysfunction and pathology have also been associated with cognitive decline, neuronal loss, Aβ and tau pathology (34, 35). In addition, cerebral amyloid angiopathy (CAA) is considered one of the pathological hallmarks of AD and has also been observed to precede Aβ and tau pathology in a preclinical study (36, 37). The finding that the tight configuration of astrocytic end-feet that surround endothelial cells is altered with Aβ deposition in capillaries also suggest a possible general impairment of astrocytic capacity for regulating blood flow, in turn likely contributing to cognitive decline (34, 38). Furthermore, this impairment of the BBB with CAA also results in increased leakage across the BBB, including increased extravasation of peripheral immune cells into the CNS which is thought to contribute to AD pathogenesis (39). For example, reduced tight junction proteins and other intercellular structural proteins in endothelial cells have been observed with CAA (40). Aβ pathology of the BBB is also associated with overexpression of adhesion molecules like vascular adhesion molecule 1 (VCAM-1) and intercellular adhesion molecule 1 (ICAM-1), facilitating the migration of peripheral leukocytes into the CNS (41).

In the same vein, astrocytic dysfunction has been implicated in the impairment of the recently described glymphatic system responsible for removing metabolic waste products including Aβ (42). The glymphatic system comprises a paravascular channel through which cerebrospinal fluid (CSF) from the subarachnoid space can flow and directly mix with interstitial fluid (ISF) in the brain parenchyma before returning as a CSF-ISF mixture to the subarachnoid space (42). As such, the glymphatic system plays an important role in removing metabolic waste products including Aβ (42). Central to this are astrocytes that regulate the flow of CSF between the paravascular spaces and the interstitium mainly *via* aquaporin 4 (AQP4) channels located mostly on astrocytic end-feet encapsulating the blood vessels (43). However, a loss of this polarization of AQP4 channels from the end-feet to the parenchymal processes instead has been observed in AD (44). It still yet remains to be elucidated if Aβ accumulation is a consequence of this polarization of AQP4 channels or if this polarization is secondary to Aβ pathology (43, 45, 46).

### Adaptive Immune System

Research into the role of the CNS adaptive immune system in AD pathogenesis has been increasing in recent years, albeit slowly, and at present this topic remains poorly understood. Post-mortem brain tissue of AD patients has been reported to show increased numbers of T lymphocytes than those of healthy controls (47). In particular, most of these cells were found to be CD8+ T cells and were significantly correlated with tauopathy but not Aβ, suggesting a role for CD8+ T cells in neurofibrillary pathology (48). Increased CD8+ T cell numbers in the peripheral blood of AD patients has been reported, and of which are comprised mostly of clonal T cells expressing T cell receptors (TCR) with affinity to Ebstein-Barr Virus (EBV) antigens (49). With the majority of the CNS T cell population originating in the peripheral circulation and entering through the BBB, it has been posited that the CNS immune response in AD might be associated with a previous herpes virus infection or a reactivation of it (50, 51). However, a meta-analysis has recently shown that current evidence supporting either of these theories is inconsistent (52). It is also worth noting that T cell clonality and responses differ between compartments of the aging brain. For example, a population of clonally expanded T cells that are distinct from those in the peripheral blood have been reported in the aged brain and produce an IFNγ response which inhibits neural stem cell (NSC) proliferation, possibly contributing to the decline in NSCs during aging (53). Additionally, CNS-specific CD4+ effector memory-type T cells have been reported to reside in the choroid plexus (CP) epithelium which exhibit a Th2-like inflammatory response in the aged brain, with increased IL-4 production and decreased IFNγ production (54). The increased levels of IL-4 have been associated with increased production of the chemokine CCL11 that is in turn associated with age-related cognitive impairments (55). The raised IL-4 levels are also thought to impair brain plasticity by contributing to the dysregulation of brain derived growth factor (BDNF) and other neurotrophic factors (54).

Th17 cells, like CD8+ T cells, have also been implicated in AD, with increased Th17 infiltration and upregulation of IL-17 and IL-22 in the hippocampus, blood and CSF being observed following the injection of Aβ into the hippocampi and induction of AD in rats (56). Additionally, IL-17 has been found to inhibit neurogenesis in the hippocampi of mice whilst genetic deletion of IL-17 promoted neurogenesis, possibly implicating Th17 cells in AD pathogenesis (57). T regulatory (Treg) cells have also been implicated in contributing to the pathogenesis of AD, albeit their exact role remains to be fully elucidated. For example, transient depletion of Treg cells have been associated with an IFNγ-dependent systemic immune response characterized by the peripheral recruitment of monocytes *via* the choroid plexus, and this process in turn was associated with increased Aβ plaque clearance and improvement in cognition in mice models of AD (58, 59). However, current studies showing conflicting evidence on the role of Treg cells as either beneficial or detrimental disallows any concrete conclusions on their contributions to AD pathogenesis (59–62).

### Inflammasome

The role of inflammasomes in AD pathogenesis is currently an area of increasing research interest. Whilst various types of inflammasomes exist, NLRP3 in particular has been implicated in AD. The NLRP3 inflammasome is a protein complex comprised of the NLRP3 sensor molecule, the adaptor protein ASC and pro-caspase-1 and is found exclusively intracellularly in microglia (63). One of its main functions is the generation of active IL-1β and IL-18 from their precursors in response to DAMPs, chiefly Aβ in AD (63, 64). NLRP3 levels have been found to be increased in AD brains (65). Of particular interest, loss of NLRP3 inflammasome activation in murine models of AD is associated with decreased Aβ deposition and IL-1β levels, and increased phagocytic activity (65). Additionally, it has also been shown that IL-1β can inhibit Aβ clearance (63). Therefore, these findings suggest that NLRP3 play a significant role in the neuroinflammatory process in AD.

### Meninges, Choroid Plexus, and Skull Bone Marrow

There has in recent years been an increasing interest and appreciation of various neuroimmune interfaces in AD pathology, namely the meninges, choroid plexus (CP) and skull bone marrow. The (re)discovery of the meningeal lymphatic system has led to the hypothesis that the dysfunction of these vessels contributes to AD pathology through impaired clearing of Aβ and consequently increased Aβ deposition (66–68). Supporting evidence for this includes the finding that macromolecules are drained into the cervical lymph nodes from the CNS *via* these lymphatic vessels, and that treatment of aged mice with vascular endothelial growth factor (VEGF) C improves drainage of macromolecules from the CSF and improves cognitive performance (68, 69). Conversely, the loss of these lymphatic vessels in transgenic mice led to increased Aβ deposition, and cognitive and behavioral symptoms (68, 69). In addition to these lymphatic vessels, the meninges serve as an important neuroimmune interface for modulating local immune responses. For example, the loss of C-C chemokine receptor (CCR) 7 in transgenic mice led to increased meningeal Treg responses, attenuated meningeal effector T cell responses, and cognitive deficits (59).

It has also recently been reported that a network of direct vascular channels linking the skull bone marrow and the meninges exists and serves as an important migratory route for the recruitment of myeloid cells into the brain (70, 71). Given the salient role of microglia in AD pathogenesis, these findings suggest that the skull bone marrow serves as an important source of peripherally-derived monocytes and microglia that contribute to the disease process (70). Additionally, it has recently been reported that the majority of myeloid cells in the meninges are derived from the skull bone marrow both in homeostatic and pathological states (72). Of note as well is the role of the skull bone marrow as a source of B cells to the meninges, with these developing B cells travelling *via* the direct vascular network to the meninges to complete their maturation (73). It is worth noting however that the role of B cells in AD pathogenesis still remains elusive. For example, in a recent study by (74), mice models lacking B cells and antibody responses were associated with the activation of endogenous retroviruses in the hippocampi which in turn was associated with memory impairments (74). Conversely, whilst B cells have been shown to produce immunoglobulins and cytokines that reduce Aβ plaques and ameliorate AD pathology, a recent study has reported an improvement in behavioral and memory symptoms with the genetic loss and transient depletion of B cells (75).

The CP is another neuroimmune interface that plays an important role in modulating the immune response in the CNS. For example, it has been shown that the CP integrates peripheral immune signals with those from the CNS, and is particularly important as a trafficking route for anti-inflammatory cells into the CNS, potentially influencing AD pathogenesis (76). It is likely that CP functioning is dependent upon a complex interplay between various cellular players including CP mesenchymal and epithelial cells, summarized by Dani et al. (77). In addition, it has recently been reported that various myeloid immune cell subset populations of microglia and dendritic cells reside in the CP and meninges and is summarized by Mrdjen et al. (78). However, much remains to be elucidated about the exact role of the CP in AD and the roles of these cells in health and disease (77, 78). Recently also the dural sinuses have been reported to be an important neuroimmune interface where CNS-derived antigens in the CSF are sampled by local antigen presenting cells (APCs) and presented to patrolling T cells (79). However as with the CP, much remains to be elucidated about its significance in various neurodegenerative pathologies including AD.

## Age-Related Changes in the CNS Immune Response

### Impaired Adaptive Immunity

Due to various age-related changes such as thymic involution, there is a decline in T cell numbers with age (80). However, more notably, there is a decrease in the number of naïve T cells and an increase in age-experienced cells, particularly the CD28-CD8+ T cell population (80, 81). These cells are only seen in the elderly compared to neonates and display an impaired functional phenotype, including a reduced proliferative response to TCR stimulation with a normal or augmented cytotoxic capacity and a resistance to apoptosis (82, 83). It is thought that these cells represent a population that has undergone terminal differentiation as a result of chronic stimulation by persistent infection with common pathogens such as cytomegalovirus (CMV) (80). This notion is supported by the finding that increased CD28-CD8+ T cell numbers are seen in patients with viral infections like CMV, and that naïve T cells are reduced in the elderly (80, 82). CD8+ T cells in the elderly generally also show significantly reduced interferon gamma (IFNγ) production and a reduced antigen-recognition repertoire by TCRs (80, 83). Therefore, the implication of this is two-fold: firstly, there is reduced adaptive immunity to previously encountered pathogens, and secondly that there is a reduced ability for the adaptive immune system to respond to novel pathogens (80). It is also possible that impaired IFN production in the elderly adversely affects the ability of the immune system to clear viral infections such as CMV and regulate neuroinflammation, resulting in a dysfunctional immune response that includes an impaired resolution of neuroinflammation by macrophages (84, 85). It is also worth noting that the clonal expansion of CD28-CD8+ T cells has been directly implicated in the increased infection rates and poor vaccine responses in the elderly (82).

CD4^+^ T cell function in the elderly is also altered but depends largely on when in the host’s lifetime naïve and memory CD4^+^ T cells are generated. Naïve CD4^+^ T cells from elderly humans and mice have been shown to display reduced responsiveness to TCR stimulation, and reduced IL-2 production and proliferation when presented with antigen compared to naïve CD4^+^ T cells from younger hosts (86). Naïve CD4^+^ T cells from elderly mice have also been shown to produce a poorer humoral immune response *via* the generation of germinal centers for B cells compared to those from younger hosts (87). Memory CD4^+^ T cells extracted from healthy elderly humans and mice have also been shown to respond normally to antigens and those generated early in life respond well to antigens over time (80). As such, it has been hypothesized that reduced memory CD4^+^ T cell function in old age might be due to their derivation from aged naïve CD4^+^ T cells that exhibit reduced clonal diversity and capacity for proliferation (80).

In addition to the impaired ability of CD4^+^ T cells to support the humoral immune response, other changes in humoral immunity are seen in the elderly. Similar to T cells, B cell production and numbers are reduced in the elderly along with reduced serum immunoglobulin levels (80, 88). The immunoglobulins produced also have altered specificities and affinities for antigens, with an increasing proportion of serum immunoglobulins in the elderly displaying specificities to autoantigens (89). These immunoglobulins are also low affinity IgM, reflecting an impaired capacity for class switching in B cells (80). This might also be a consequence of altered proportions of B cell subpopulations and an impaired ability for germinal center generation secondary to an impaired CD4^+^ T cell function (80).

### Impaired Innate Immunity

One key characteristic of aging microglia is their propensity for pro-inflammatory cytokine production (90). Several mechanisms have been proposed for this phenotype. Firstly, age-related neuronal damage may result in a loss of inhibitory signals to microglia (90). Secondly, the accumulation of misfolded proteins like Aβ as a consequence of normal aging promotes a pro-inflammatory response as described earlier. Finally, chronic exposure to increased transforming growth factor β (TGFβ) might impair microglial capacity for anti-inflammatory cytokine production, possibly through the downregulation of transcription factors important for switching to an anti-inflammatory phenotype (90). Aging microglia have also been demonstrated to show reduced phagocytic activity for Aβ and neuronal waste products like myelin debris (91, 92). In addition, aging microglia show reduced chemotactic capacity in response to neuronal damage (93).

## Neuroinflammation and Th1 Skewing of the Immune Response in AD

Given the age-related changes in the CNS immune system and the pathogenesis of AD described earlier, it seems that neuroinflammation and Th1 skewing of the immune response play a significant role in the pathogenesis of AD. Two dominant phenotypes of CD4+ T cells are the Th1 and Th2 types. Th1 cells are largely responsible for cytotoxic cell-mediated pro-inflammatory responses through coordinating other players such as CD8+ T cells and are essential for clearing intracellular infections (eg. viruses) and malignant cells. Th2 cells are largely responsible for humoral immunity through coordinating antibody generation by B cells (94). As described earlier, there is an increased number of CD8+ T cells, particularly CD8+ T CD28- cells, in the brains of AD patients (80). Memory CD8+ T cells can contribute to the control of CNS infections (95, 96). However, it remains unknown whether and how each subset contributes to CNS immunity. In addition, the presence of these cells positively correlates with the poor antibody response to the influenza vaccine in the elderly (95, 97). These cells also appear to produce high levels of IFNγ and low IL-5 levels, and promote a Th1 cytokine response by CD4+ T cells in response to auto antigenic stimulation (95). It is postulated that the chronic and ubiquitous exposure to autoantigens underpin the prevalence of these CD28-CD8+ clones and the high IFNγ levels in old age (95). Of note as well are the reduced rates of various cancers in AD patients, lending strength to the notion of Th1 skewing in AD pathology (98). In a mouse model of AD, the peripheral administration of antibodies against Aβ has also been shown to induce clearance of Aβ and reduce Aβ burden, suggesting that reduced antibody generation in the elderly might contribute to AD pathogenesis (99). This Th1 skewing of the immune response, namely the production of IFNγ and the reduced antibody production, could result in increased activation of microglia (94). IFNγ working in concert with TNF also triggers Aβ production in neuronal and extra neuronal cells and increased reactive oxygen species (ROS) production by microglia, likely perpetuating AD pathogenesis (80).

**Figure 1:**
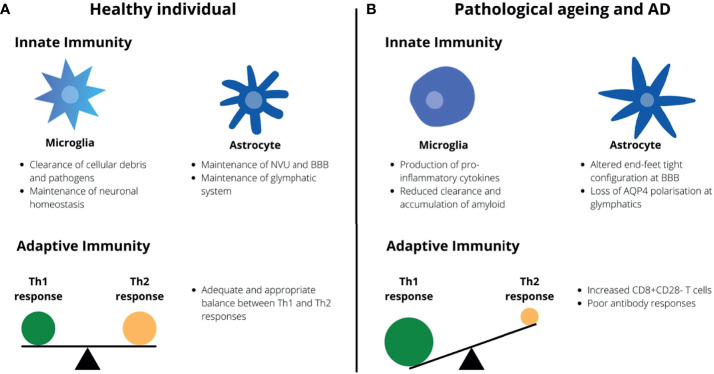
Healthy aging *vs*. pathological aging and AD. **(A)** In healthy individuals, microglia serve to provide neurotropic support and maintain neuronal homeostasis whilst astrocytes maintain the BBB and glymphatic system. There is also an appropriate balance between Th1 and Th2 responses by the adaptive immunity. **(B)** In AD and aging, microglia undergo morphological changes and adopt a pro-inflammatory phenotype whilst the capacity for astrocytes to maintain the BBB and glymphatic system is impaired. There is a Th1-skewing of the adaptive immune response with an accumulation of CD8+CD28- T cells.

There is a recent concept of microglial priming where previous and chronic disturbances in the CNS environment pushes microglia to a phenotype with more marked pro-inflammatory responses than the transient activated phenotype adopted during brief episodes of physiological insults (100). These insults include age-related oxidative stress and DNA damage, traumatic brain injuries (TBI) and CNS infections that ultimately induces a state of neuroinflammation required for microglial priming (100). With respect to microglial phenotypes, they are typically described *in vivo* as having either a pro-inflammatory activated phenotype associated with increased ROS and pro-inflammatory cytokine production and reduced neurotropic factor production, or a non-activated phenotype associated with increased anti-inflammatory cytokine production (eg. IL-10, TGFβ) and phagocytic activity (100, 101). In the case of primed microglia, these cells undergo morphological and functional changes when exposed to chronic stimulation to resemble a pro-inflammatory activated phenotype (102). However, they are characterized by a higher baseline production of inflammatory markers and mediators, a lower threshold for activations and switching to a pro-inflammatory state, and an exaggerated inflammatory response (102). The state of chronic neuroinflammation that follows can lead to the loss of neurons and the development and deposition of neurofibrillary tangles (NFT) and Aβ (100, 102). This as such might lead to a self-perpetuating cycle of Aβ deposition secondary to the accumulation of primed microglia which in turn stimulate a pro-inflammatory response by microglia. In addition, the reduced clearance of Aβ by mechanisms such as reduced microglial phagocytic capacity, increased IFNγ production and reduced antibody generation in the elderly may further exacerbate this neuroinflammation towards symptomatic AD (**Figure 1**). Comparisons between the immune system of a healthy individual and that of the elderly and AD patients are summarized in **Figure 1**.

There is evidence however that the neuroinflammatory process differs throughout the time course of AD pathogenesis. In a study by Vlad et. al., the risk of developing AD gradually reduced in proportion to the duration of NSAID therapy, reaching to levels almost half that of the general population after a few years (103). Subsequent studies have found similar results (104, 105). However, a Cochrane study has found no significant therapeutic benefit of anti-inflammatory drugs in the treatment of symptomatic AD (106). Supporting this notion of a changing neuroinflammatory process is the finding that oxidative damage is greatest early in AD and decreases with disease progression (80). It has been suggested as such that as AD worsens, Aβ deposition increases alongside compensatory changes to reduce oxidative damage, which might include dysregulated T cell responses (80).

**Figure 2:**
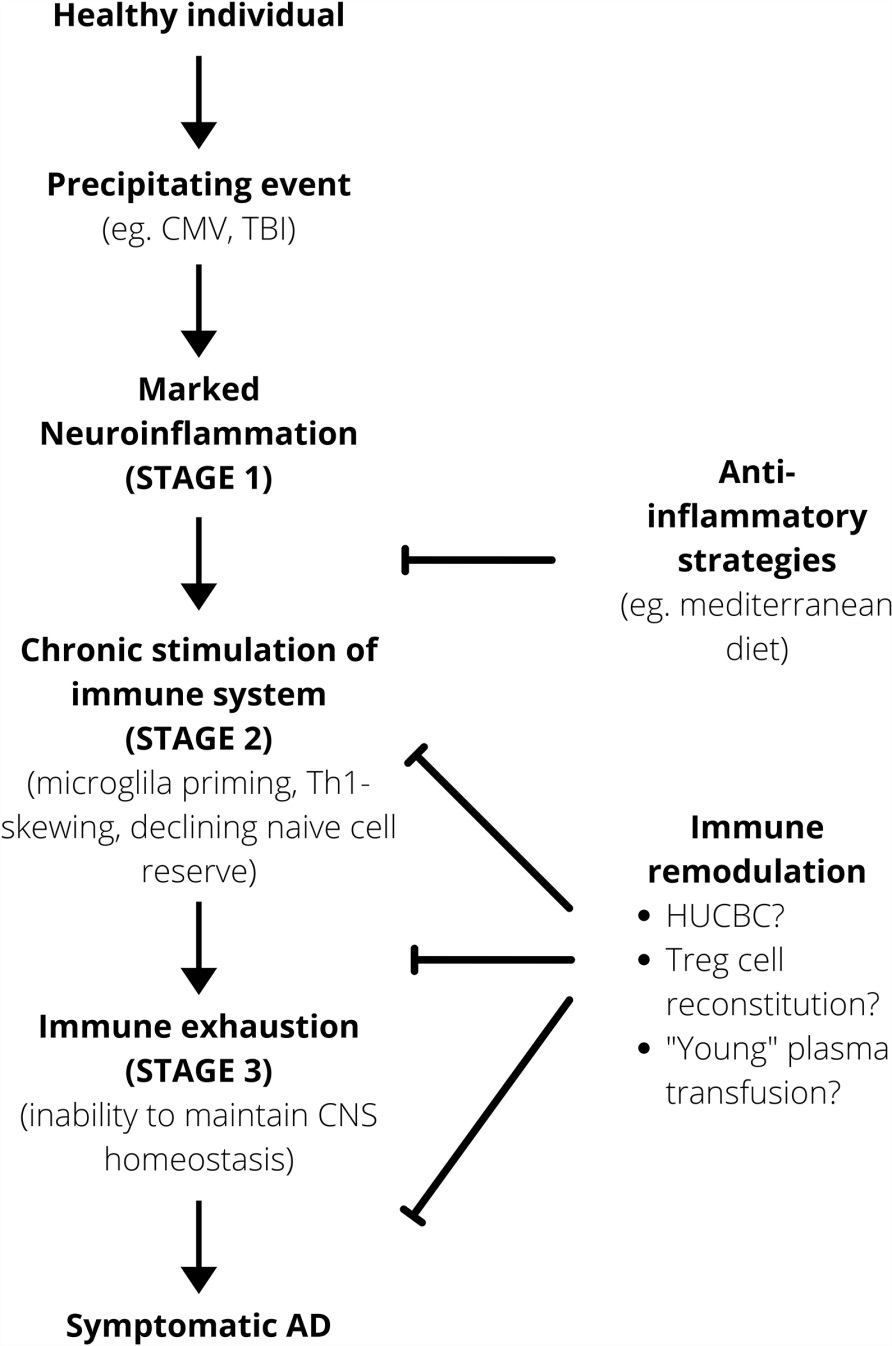
Working model for AD pathogenesis. Anti-inflammatory strategies are important prophylactically to minimize that risk of the immune system progressing onto a self-perpetuating path towards immunosenescence. After a certain point, immune remodulation is required to alter the disease course. HUCBC, Human Umbilical Cord Blood Cell.

It seems therefore that the pathogenesis of AD could potentially be divided into three broad stages. In the first instance, there is a period of marked neuroinflammation following physiological insults. This then progresses to the second stage characterized by a self-perpetuating state of chronic stimulation and neuroinflammation that potentially promotes the accumulation of primed microglia and a pro-inflammatory environment. This chronic neuroinflammation and stimulation, caused either by a transient and brief insult like a TBI or by chronic infection by viruses like CMV, pushes the CNS immune system towards immunosenescence, the third stage and final stage, where its ability to adequately maintain the homeostatic neuronal environment is impaired (**Figure 2**). Cellular senescence is characterized by the inability of cells to proliferate, this process being related to the erosion of telomere (107). Moreover, senescence is characterized by 3 main phases: the induction phase (characterized by telomere shortening, DNA damage, growth factor deprivation); the second phase – DNA damage response (characterized by DNA damage response); and phase 3 – growth arrest (signal transduction molecules p53, p21 trigger growth arrest). However not all primary cells senesce in the same way, for instance fibroblasts present a pre-senescence phase that may be reversible. In contrast T cell senescence does not seem related to shortening of telomere, and they die when they differentiate (107). Whatever the mechanism is, aging (p53 up regulation, mitochondrial dysfunction, DNA damage) and inflammation represent a two-way system in neurodegeneration.

Post-mortem brains of patients without a history of dementia have also been found to have sufficient Aβ and NFT amounts that would have otherwise prompted a diagnosis of AD, supporting the role of a dysfunctional CNS immune response in determining the onset of symptomatic AD (80, 108). One population that has been studied particularly with regards to health in old age are centenarians. Various anti-inflammatory mechanisms such as increased cortisol and IL-10 levels are well documented (80). This might enable centenarians to suppress neuroinflammation in the early stages before chronic neuroinflammation and significant CNS immune dysfunction develops. Interestingly, it has also been observed that administration of IFNβ-1, an antiviral agent, in patients with early AD for twenty-eight weeks resulted in significant improvements in some cognitive functions but not in inhibiting the disease progression (109). This raises the possibility of early antiviral treatment as a potential novel therapeutic option in early AD (109). It is also worth noting that whilst the role of Treg cells in AD pathogenesis remain elusive, centenarians have been noted to have increased numbers of Treg cells and that this positively correlated to improved survival outcomes (58, 110). It is possible that Treg and anti-inflammatory responses may differ significantly between mice models of AD and AD in humans in a manner that is currently poorly understood.

## Anti-inflammatories and Aβ Vaccines as Therapeutic Options for AD

Given the association between Aβ and AD, trials exploring the therapeutic potential Aβ vaccines have been conducted. The first attempt at developing an Aβ vaccine was by Schenk et al. who prepared a vaccine from Aβ_1-42_ peptide and Freud’s adjuvant (111). When tested on murine models of AD, the vaccine was found to confer both significant prophylactic and therapeutic benefits in the prevention of Aβ plaque formation and in inhibiting the progression and extent of Aβ deposition respectively (111). This vaccine was then developed for clinical trials but did not progress beyond phase II due to a proportion of patients developing aseptic meningoencephalitis (112). In a follow-up study on patients from this initial trial, post-mortem analysis of the brains of eight of the patients who had received the vaccine and had died with AD showed significant variation in Aβ burden. More notably however was the finding that in seven of the eight vaccinated patients including those with virtually complete Aβ plaque removal there was a severe end stage dementia prior to death (113). Although it is not possible to fully ascertain the therapeutic potential of therapies targeting Aβ from this study alone due to the small sample size analyzed post-mortem in the follow-up and the adverse events associated with the vaccine, it is possible however that future attempts at relieving Aβ burden may eventually reap limited rewards. This lack of association between Aβ burden and the clinical course of AD has been supported by other studies showing similar findings (108, 114). Attempts at using human intravenous immunoglobulins (IVIG) on the basis of preclinical findings showing Aβ clearance by IVIG have also failed to produce significant cognitive improvement in patients. Interestingly, however, was the finding that IVIG significantly reduced Aβ levels and slow cognitive decline in patients with the ApoE4 allele, although cognitive decline was only observed on neuropsychiatric examination with no corresponding improvement in activities of daily living (ADL) (115, 116). As such, it is possible that therapies targeting Aβ clearance may only prove efficacious for a small group of AD patients.

As described earlier, current evidence suggests a role for anti-inflammatory therapies in AD particularly as prophylaxis and treatment during the prodromal stage. At present, there is increasing interest in the inflammasome, particularly NLRP3, as a novel target for anti-inflammatory therapy research in AD. The NLRP3 inflammasome inhibitor dapansutrile (OLT1177) has been investigated for its therapeutic potential in other inflammatory conditions, having recently completed a phase II trial in treating osteoarthritis and acute gout flare (117). Given the association between NLRP3 and Aβ and pro-inflammatory responses in AD, dapansutrile has recently been investigated pre-clinically for the treatment of AD (118). In this study, dapansutrile was shown to reverse cognitive impairment, reduce microglial activation and cortical plaques, and normalize plasma metabolic markers of AD in murine models (118).

Since the success of the anti-TNF therapy infliximab in the treatment of Crohn’s disease, there has been a considerable interest in the following years in developing therapies targeting specific cytokines for various inflammatory conditions (119). One such drug currently of interest in the treatment of AD is etanercept. Similar to infliximab, etanercept is a TNF inhibitor. Various trials conducted have corroborated the positive therapeutic potential of etanercept in treating AD (120). However, in a recent phase II trial, although etanercept was found to be generally well tolerated by patients there was no statistically significant changes in cognition, behavior or global function (121). Interestingly, one mechanism by which etanercept might improve AD symptoms and neuropathology if any is that it modulates TNF regulatory function of synaptic transmission (120).

Naturally occurring anti-inflammatories and the role of certain diets have also been explored for their therapeutic potential in AD. One particular diet well-known for its anti-inflammatory properties is a Mediterranean diet (MD). This diet is characterized by large intake of fruit, vegetables, wholegrains, nuts and legumes; moderate consumption of fish, poultry and alcohol, and restricted intake of red and processed meats alongside olive oil as the main source of fat (122). Observational studies have found that greater adherence to the MD is associated with improved cognitive performance, slower cognitive decline and reduced risk of cognitive impairment and AD (122). The MD has also been associated with protective brain structures and function such as increased cortical thickness, greater brain volumes, reduced hippocampal atrophy rate and reduced Aβ accumulation (122). However, it seems that adherence to the MD for at least a few years is required to reap the cognitive benefits. In the PREDIMED study, adherence to the MD over 4-6 years was particularly associated with improved global cognition, memory and executive function (122). However, no cognitive benefits were seen in populations on the MD for up to one year, although improved global cognition and episodic memory were seen in those with greater adherence to the diet (123–125). These findings are not surprising as they reflect the findings of trials investigating the use of NSAIDs for treating AD as described earlier (103–106). It would be interesting however to explore in greater detail the role of a MD in managing symptomatic AD as it may be possible that there may be other beneficial compounds that may influence the disease course either through regulating neuroinflammation or by other pathways.

## Rethinking the Role of Aβ in AD

The role of Aβ in AD pathogenesis was first proposed as part of the ‘amyloid cascade’ hypothesis following the observation that virtually all people with Down’s Syndrome (DS) will exhibit AD pathology by 40 years of age, and that there was concomitant overexpression of Aβ from the cloning of the *APP* gene with trisomy 21 (126). *APP* cloning and subsequent overexpression of Aβ also causes familial EOAD (127). However, as described earlier, post-mortem studies showing a lack of correlation between Aβ levels and clinical AD, and the current lack of therapeutic success in therapies targeting Aβ suggest that a dysfunctional immune response might play a more significant role in AD pathogenesis. In fact, people with DS typically have immune systems that are not dissimilar to that seen in AD and old age. For example, poor vaccine responses are frequently observed in DS patients alongside decreased immunoglobulin levels and B cell populations (128). Increased Th1/Th2 ratio with corresponding decreased risk of various cancers, decreased T cell proliferation and reduced naïve T cell populations are also seen (128, 129). Therefore, it is possible that a dysfunctional immune system might be the main player in the pathogenesis of AD, with Aβ likely exacerbating the process but not being a causative agent. For example, the observation that Aβ deposition is initially diffuse with gradual progression to neuritic plaques but accumulating at an exponential rate after 40 years of age in DS patients might be due to an earlier initiation and acceleration of the process driving the CNS immune system towards a state of immunosenescence resembling that seen in AD patients (126). Similarly, it is possible that overexpression of amyloid such as is seen with DS patients might be a key player driving the progression towards immunosenescence. Interestingly as well is the finding that DS individuals are more sensitive to IFN stimulation due to the overexpression of its receptor located on the extra chromosome 21 (130). It is possible that this might lead to increased activation of microglia and ROS production that in turn accelerates aging and depletion of immune reserves *via* chronic inflammation (131)

It has also recently been proposed that Aβ might be an innate effector molecule (132). For example, Aβ has been shown to have antimicrobial properties against viruses, bacteria and fungi and enact this by forming oligomers (133). In addition, overexpression of Aβ provides increased resistance to bacterial and viral infections (134). Aβ has also been proposed to functionally resemble that of cytokines and should be considered as such (132). This theory might account for the close spatial and temporal association between microglia and Aβ frequently observed in AD. It also strengthens the argument that AD might be brought on by CNS immunosenescence secondary to chronic stimulation like persistent CMV infection as the CNS immune system attempts to clear the infection (80).

## Novel Strategies for Remodulating the CNS Immune Response

Human umbilical cord blood cells (HUCBC) have been garnering increasing interest as a potential strategy for remodulating the CNS immune response amid observations that HUCBC inhibits Th1 whilst promoting Th2 responses in murine models of stroke and neurodegenerative diseases (80, 135, 136). Hippocampal administration of HUCBC in murine models of AD have also been demonstrated to reduce neuropathological changes and improve cognitive impairment (137). Notably, there was a reduction in Aβ deposits and tau hyperphosphorylation, improved spatial learning and memory, a switching in cytokine profile exhibited by microglia from a pro-inflammatory phenotype to an anti-inflammatory one, and that these alternatively activated microglia were associated with Aβ (137). Such findings have led to the undertaking of a phase I clinical trial involving HUCBC mesenchymal stem cells (MSC) administration into the hippocampus of nine patients with mild to moderate AD (138). This method was found to be well tolerated and safe, although the efficacy was not able to be properly assessed in this study (138). Subsequent clinical trials have corroborated the safety profile of HUCBC-MSC administration into the brain. However, clinical outcomes have been less encouraging, with no significant improvement in cognition being reported (139). These clinical trials and their findings are summarized by Liu and colleagues (139). In addition to various technical issues such as the specific cell stage to be transplanted that remain to be resolved, it has been posited that the lack of clinical success with stem cell therapy research for AD could be due to inappropriate timing of stem cell transplantation (139). Although CSF biomarkers have strong predictive value in diagnosing or excluding AD in patients with mild dementia, individual clinical courses tend to be more variable which may reflect different stages and severity of neuropathology that may render subsequent stem cell transplantation ineffective (139).

Another strategy that might be useful to explore would be the reconstitution of Treg cells in AD patients. It has recently been shown that Treg anti-inflammatory function is impaired in patients with AD, but had increased suppressive activity on effector T cell proliferation and macrophage activation following *ex vivo* clonal expansion (140). This suggests that Treg function and immunophenotype are impaired in AD, but that administration of *ex vivo* clonally expanded of Tregs might be a viable therapeutic option (140). More recently, *in vivo* expansion of Tregs has been increasingly explored as a preferred method to *ex vivo* expansion, one main reason being that it bypasses the major obstacle of low Treg frequencies in lymphoid tissues that as such requires *in vitro* expansion prior to adoptive transfer (140). *In vivo* expansion of Tregs can be achieved *via* administration of low-dose IL-2 (141). IL-2 therapy has been shown to reduce Aβ load and improve neuroplasticity that was associated with recovery of memory deficits (60, 141). However, due to the short t_1/2_ of IL-2, one method that may help to overcome this is the co-administration of IL-2 with an IL-2 specific monoclonal antibody, such as JES6-1A12, which increases its t_1/2_ (141).

Findings such as the functional recovery of Tregs following *ex vivo* expansion in AD patients suggest that strategies tailored toward alteration of the AD microenvironment may help restore immune balance. One novel strategy could be in the form of transfusions of plasma or specific plasma constituents from young donors to elderly or AD recipients. Murine models of plasma transfusion including parabiosis studies have shown improvement in both neuropathological changes with reduction of Aβ and tau burden and improvement in cognition (142, 143). Various clinical trials exploring different types of therapeutic plasma exchange (TPE) in patients with established AD have generally yielded promising results, the findings of which are summarized by Imbimbo and colleagues (144). However, concrete conclusions are difficult to draw at this stage due to the inherent limitations of the study designs such as small sample sizes and strict exclusion criteria (144). It is also worth noting that one reason for the particular interest in TPE over other blood products such as whole blood might be due to previous associations found between blood transfusions and an increased risk of dementia or AD (145).

Whilst it is currently unclear how TPE with plasma from young donors might exert their therapeutic effects, several theories have been proposed. Vascular cell adhesion molecule 1 (VCAM1), an important molecule in mediating attachment of immune cells to the endothelial surface, has recently been found to be overexpressed in the brains and plasma of both aged mice and humans (146). In addition, systemic administration of anti-VCAM1 immunoglobulins and deletion of the *Vcam1* gene in brain endothelial cells (BECs) was shown to reverse microglial reactivity and cognitive defects in mice (146). Therefore, it has been posited that increased inflammatory cytokine levels in old plasma increase VCAM1 expression on BECs, resulting in peripheral leukocyte recruitment and BEC inflammation, in turn activating microglia (146). Another theory for the mechanism of TPE in AD is that old and young plasma may contain youthful and pro-aging factors that influence that ultimately influence the CNS environment and immune response (142). Just as old plasma may contain higher levels of pro-aging pro-inflammatory cytokines, young plasma may contain factors that promote healthy aging of the CNS (142). For example, systemic administration of growth differentiation factor 11 (GDF11) was shown to increase neural stem cell numbers and vascular remodeling in the hippocampi of aged mice (142). However, more research is required to identify these factors in old and young plasma before specific supplementation with these factors can be considered as a viable therapeutic approach for AD.

Given the qualitatively and quantitatively poor production of immune cells seen in the elderly and in AD patients, bone marrow transplantation might be a potential therapy for AD. It has been found, for example, that transplantation of a young bone marrow into old recipient mice preserved cognitive functions, reduced microglial activation in the hippocampus and preserved synaptic connections (147). However, even if bone marrow transplantation was demonstrated to be highly effective in treating AD, the practical limitations in implementation are likely to exclude it as a treatment offered by healthcare systems. These may include availability of compatible bone marrows for transplantation and ethical considerations over which AD patients should receive it and if they should receive them at all in preference over other patient groups like childhood hematological cancers to name a few. Perhaps a more feasible approach might be strategies aimed at preserving and restoring thymic function. Various strategies to rejuvenate thymic function currently being explored are broad and diverse, and are discussed in detail in a review by Thomas and colleagues (148). It should also be noted, however, that in addition to these novel therapies simple strategies such as regular physical exercise have also been shown to have prophylactic and therapeutic benefits in reducing the risk of AD and improving cognitive decline in AD patients (149). The means through which they exert their beneficial effects include the slowing of age-related deterioration in thymic output and decreasing pro-inflammatory cytokine levels associated with neurodegeneration or “inflammaging” (150).

## Biomarkers for AD

Identifying suitable biomarkers for monitoring different stages of the AD pathogenic course would be important for future research into therapeutic options for AD. Various biomarkers directly relating to the changing immune response in the AD continuum have been explored, one example being the blood compound neopterin. Neopterin is produced by IFNγ-stimulated macrophages and its association with cell-mediated responses has made it a common biomarker for monitoring such processes (80, 151–153). Notably, neopterin levels are increased in AD patients compared to age-matched controls and have been suggested to reflect serum immunity to CMV (80). In addition, neopterin levels were significantly associated with cognitive decline in AD patients (151). However, whilst neopterin levels were significantly higher in AD relative to MCI patients, there has been no significant difference in neopterin levels between MCI patients and cognitively normal individuals (154). Significantly increased neopterin levels were also only observed in patients with moderate dementia as assessed by their mini mental state score (MMSE) (153). This suggests that the usefulness of neopterin as a biomarker for AD is restricted to patients with established AD and is unlikely to be useful for this purpose during the preclinical phase for screening.

It is well acknowledged that immune changes are seen in the peripheral blood of AD patients. There is growing evidence showing peripheral immune changes in AD such as reduced CD4+ T cells and regulatory T cells (155). However, although T cell and B cell numbers and function have been shown to be altered in AD, there is a great amount of discordance between the results which are summarized in the review by Martorana et al. (155). The cytokine profile in the peripheral blood has also been documented reflecting the pro-inflammatory state associated with AD and are discussed in a review by Park and colleagues (156). Whilst attempts to accurately profile peripheral immune changes in AD may eventually lead to the discovery of new biomarkers, current understanding of these changes does not support their usefulness as biomarkers for monitoring the pre-symptomatic stage of AD. This is mainly due to virtually all current research into this being focused on patients with established AD. In addition, as discussed earlier, whilst a general state of neuroinflammation might predominate earlier in the AD continuum, the skewing of the immune response might be occurring much later in the course. It has also been found that the time spent in each phase of the continuum varies between individuals influenced by various factors (157). Essentially, what then remains to be more adequately understood is what the threshold is for pushing the immune system onto the self-perpetuating chronic inflammatory state towards immunosensence and what are the events that can precipitate this process. As discussed previously, events like CMV infection can lead to a state of chronic immune stimulation that depletes the limited immune reserves. This process of inflammaging is thought to play a key role in determining healthy and pathological aging (158). Better understanding and recognition of this threshold could help to identify patients for early intervention and produce better outcomes in old age.

It should be noted however that one main obstacle to research into peripheral immune changes in individuals with AD is the likely unwillingness of AD patients and their relatives to participate in the study given the substantial challenges already being experienced by them from the disease. This might be circumvented to some extent by gaining consent for post-mortem analysis of immune changes in the tissues, which might also have an added advantage of any findings directly reflecting the pathogenic process compared to peripheral blood immune changes which may differ. However, delays in acquiring and analyzing the samples and processing of the samples may alter the samples and potentially produce misleading findings. Alternatively, it might prove useful to further investigate the skull bone marrow especially for its therapeutic potential. The skull bone marrow provides a rapid source of immune cells to the brain parenchyma *via* direct vascular networks, which have been found in both mice and humans. Additionally, given the lack of a BBB in the dura mater, immune processes and cells may be more easily targeted by therapeutics and findings in mice models may be more easily translatable to humans (159).

Current attempts at producing a dementia risk assessment tool have begun making its way into clinical trials. For example, the Cardiovascular Risk Factors, Aging and Incidence of Dementia (CAIDE) score that takes into account risk factors like age, hypertension, hypercholesterolaemia, physical inactivity, obesity and educational attainment has demonstrated cognitive benefits in at-risk individuals identified by their CAIDE score following a multimodal intervention in the FINGER trial (160, 161). However, it may be possible that such risk scores may be limited in their effectiveness given the significant proportion of the population who may present with such a constellation of risk factors (162). This might make the large-scale implementation of multimodal interventions such as that used in the FINGER trial impracticable. It might prove more useful in future to design a risk assessment tool that factors in known precipitants and biomarkers of inflammaging such as CMV seropositivity and previous TBI to better target interventions to individuals that may already be progressing towards immunosenescence and AD.

## Conclusion

There is good evidence to suggest that AD might be a consequence of CNS immunosenescence secondary to chronic inflammaging. The lack of therapeutic success of Aβ-directed and anti-inflammatory therapies alongside the recent discovery of novel properties of Aβ also prompts for a reconsideration of the role and function of Aβ, from being an etiological factor in AD pathogenesis to an innate immune response mediator. It is also important to note that whilst animal models have aided in our understanding of AD, there is still an unmet need for more humanized *ex vivo* models of the disease. This is due to the limitations intrinsic to current models such as 5XFADs that present an accelerated form of aging that does not occur in humans, in addition to differing brain areas and gene signatures between humans and the animal model. It is possible that single RNA sequencing may help in defining better understanding the disease. Additionally, characteristic AD features such as tau pathology may prove more useful as biomarkers rather than as potential therapeutic targets. However, future research into identifying new biomarkers especially in the preclinical stage of AD may aid development of early therapeutic interventions that may alter its clinical course.

## Author Contributions

SEJC performed the scientific literature search, design the figures and wrote the review. ES supervised, completed and finalized the review. All authors contributed to the article and approved the submitted version.

## Funding

This work was supported by Alzheimer’s Research UK (Pilot Grant ARUKPPG2013B-2 to ES), Fondazione Italiana Sclerosi Multipla-FISM to ES (2014/R/21). And research contract grant number TMTL1DR to ES.

## Conflict of Interest

The authors declare the absence of any commercial or financial relationships that could be construed as a potential conflict of interest.

## Publisher’s Note

All claims expressed in this article are solely those of the authors and do not necessarily represent those of their affiliated organizations, or those of the publisher, the editors and the reviewers. Any product that may be evaluated in this article, or claim that may be made by its manufacturer, is not guaranteed or endorsed by the publisher.
